# Draft genomes of two *Vibrio* spp. isolated from the brown alga *Sargassum ilicifolium* in Singapore

**DOI:** 10.1128/mra.00328-25

**Published:** 2025-05-20

**Authors:** Hau Ching Luk, Jessica A. Taylor, Deborah Yebon Kang, Su Xuan Gan, Prasha Maithani, Joao P. A. Pereyra, Yann F. Boucher, Rebecca J. Case

**Affiliations:** 1 Singapore Centre for Environmental Life Sciences Engineering (SCELSE), Nanyang Technological University 54761, Singapore, Singapore; 2 School of Biological Sciences, Nanyang Technological University 219572, Singapore, Singapore; 3 Singapore Centre for Environmental Life Sciences Engineering (SCELSE), National University of Singapore 37580, Singapore, Singapore; 4 Saw Swee Hock School of Public Health, National University Singapore and National University Health System 203377 , Singapore, Singapore; 5 Infectious Diseases Translational Research Program, Department of Microbiology and Immunology, Yong Loo Lin School of Medicine, National University of Singapore and National University Health System 63751 , Singapore, Singapore; Wellesley College, Wellesley, Massachusetts, USA

**Keywords:** *Vibrio parahaemolyticus*, *Vibrio alginolyticus*, brown alga, *Sargassum*, vibriosis, type III secretion system, hemolysin

## Abstract

*Vibrio parahaemolyticus* SSi068 and *Vibrio alginolyticus* SSi062 were isolated from the brown macroalga, *Sargassum ilicifolium*, in Singapore. Both genomes encode a type III secretion system (T3SS) and thermolabile hemolysin but lack genes for thermostable direct hemolysin (TDH) and TDH-related hemolysin. Their isolation suggests that macroalgae are a habitat for pathogenic *Vibrios*.

## ANNOUNCEMENT

*Vibrio parahaemolyticus* and *Vibrio alginolyticus* are halophilic Gram-negative bacteria often associated with seafood (1) and are an increasingly significant cause of bacterial gastroenteritis in both Asia and the United States (2, 3). Their isolation from *Sargassum ilicifolium* suggests macroalgae are a potential niche for these pathogenic *Vibrio* species. Their genomes contain key virulence factors, providing insight into pathogenic mechanisms in environmental isolates.

*S. ilicifolium* thalli (5–6 cm long × 2.5–3 cm wide) were collected from St. John’s Island (1.222353 N 103.848702 E) during low tide and transported to laboratory in sterile seawater. Thalli were heat-treated at 55°C for 6 min in sterile seawater, then stamped onto marine broth (Difco 2216) agar plates. For each isolate, a single colony was inoculated into marine broth and streaked onto marine agar plates, both incubated at 30°C. Biomass from the cultures was combined after 24 h of aerobic growth and washed with phosphate-buffered saline for DNA extraction. DNA was extracted with the DNeasy Blood & Tissue Kit (Qiagen, Hilden, Germany), with the addition of RNase A (Qiagen). DNA library preparation and sequencing were performed by the SCELSE Sequencing Facility, NTU. DNA library preparation was done using the TruSeq Nano DNA Library Prep Kit (Illumina, San Diego, USA). Sequencing was performed using an Illumina HiSeq X Ten platform, v2.5, generating 150 bp paired-end reads.

Adaptor trimming and quality filtering of raw reads were performed using Trimmomatic v0.39 (4), and genomes were assembled (Table 1) through the Shovill pipeline v1.1.0 (https://github.com/tseemann/shovill) with SPAdes v3.15.5 (5). No plasmids were found with PlasmidFinder v2.0.1 (6). Default parameters were used for all bioinformatics software described above.

Taxonomic classification of SSi062 (for Singaporean *S. ilicifolium* isolate) as *V. alginolyticus* (GCF_000354175.2) and SSi068 as *V. parahaemolyticus* (GCF_001558495.2) was determined using the Genome Taxonomy Database Toolkit (GTDB-Tk) v1.7.0 (7). Average nucleotide identity (ANI) was calculated using the orthologous average nucleotide identity, v0.93.1 (8), and DNA-DNA hybridization (DDH) score was calculated with the genome-to-genome distance calculator, v3.0 (9). The two genomes showed <95% ANI and <70% DDH with each other, but >95% ANI and >70% DDH with their respective reference genomes.

Using GhostKOALA software v3.1 (10), genes encoding a type III secretion system (T3SS) were detected in both genomes. Homologues of *tdh* and *trh* were absent from the genomes when searching with the NCBI Web tBLASTx (11). Environmental isolates with a T3SS have been implicated to be cytotoxic toward human gastrointestinal cells (12, 13). Thermostable direct hemolysin (TDH) and TDH-related hemolysin (TRH) are key virulence factors in clinical strains of both *V. parahaemolyticus* and *V. alginolyticus* (14–16), and their absence indicates that these isolates are less likely to display high virulence toward humans. Another virulence marker, thermolabile hemolysin, usually associated with infectious strains displaying lower virulence (17), was present in both genomes. Ampicillin resistance was detected in both genomes using Staramr v0.9.1 (18). The isolation of *V. parahaemolyticus* SSi068 and *V. alginolyticus* SSi062 reveals *S. ilicifolium* as a potential niche for pathogenic *Vibrio*s in tropical coastal systems.

**TABLE 1:** Genome features and GenBank accession numbers of the two isolates

Isolate	Genome size (bp)	No. of CDS^*a*^	No. of rRNAs	No. of tRNAs	G+C content (%)	No. of contigs	N_50_ (bp)	L_50_	Assembly accession no.	SRA accession no.	No. of reads	Average read length (bp)
*V. alginolyticus* SSi062	5,078,899	4,509	25	111	44.9	95	489,764	4	JBMIHU000000000	SRR32704516	7,157,055	151
*V. parahaemolyticus* SSi068	5,085,804	4,563	20	117	45.4	81	543,987	3	JBMIHT000000000	SRR32704515	6,482,905	151

^*a*^ CDS, coding DNA sequences.

## Data Availability

The accession numbers for genome assembly and SRA data, deposited to NCBI and GenBank, respectively, are provided in Table 1.
